# The Role of Thyrotropin Receptor Activation in Adipogenesis and Modulation of Fat Phenotype

**DOI:** 10.3389/fendo.2017.00083

**Published:** 2017-04-19

**Authors:** Mohd Shazli Draman, Michael Stechman, David Scott-Coombes, Colin M. Dayan, Dafydd Aled Rees, Marian Ludgate, Lei Zhang

**Affiliations:** ^1^Thyroid Research Group, Cardiff University, Cardiff, UK; ^2^Department of Endocrine Surgery, University Hospital of Wales, Cardiff, UK; ^3^School of Medicine, Neuroscience and Mental Health Research Institute, Cardiff University, Cardiff, UK

**Keywords:** adipogenesis, thyrotropin receptor, body composition, white adipose tissue, BRITE adipocytes, brown adipose tissue

## Abstract

Evidence from clinical and experimental data suggests that thyrotropin receptor (TSHR) signaling is involved in energy expenditure through its impact on white adipose tissue (WAT) and brown adipose tissue (BAT). TSHR expression increases during mesenchymal stem cell (MSC) differentiation into fat. We hypothesize that TSHR activation [TSHR*, elevated thyroid-stimulating hormone, thyroid-stimulating antibodies (TSAB), or activating mutation] influences MSC differentiation, which contributes to body composition changes seen in hypothyroidism or Graves’ disease (GD). The role of TSHR activation on adipogenesis was first investigated using *ex vivo* samples. Neck fat (all euthyroid at surgery) was obtained from GD (*n* = 11, TSAB positive), toxic multinodular goiter (TMNG, TSAB negative) (*n* = 6), and control patients with benign euthyroid disease (*n* = 11, TSAB negative). The effect of TSHR activation was then analyzed using human primary abdominal subcutaneous preadipocytes (*n* = 16). Cells were cultured in complete medium (CM) or adipogenic medium [ADM, containing thiazolidinedione (TZD), PPARγ agonist, which is able to induce BAT formation] with or without TSHR activation (gain-of-function mutant) for 3 weeks. Adipogenesis was evaluated using oil red O (ORO), counting adipogenic foci, qPCR measurement of terminal differentiation marker (*LPL*). BAT [*PGC-1α*, uncoupling protein 1 (*UCP1*), and *ZIC1*], pre-BAT (*PRDM16*), BRITE− (*CITED1*), or WAT (*LEPTIN*) markers were analyzed by semiquantitative PCR or qPCR. In *ex vivo* analysis, there were no differences in the expression of *UCP1, PGC-1α*, and *ZIC1*. BRITE marker *CITED1* levels were highest in GD followed by TMNG and control (*p* for trend = 0.009). This was associated with higher WAT marker *LEPTIN* level in GD than the other two groups (*p* < 0.001). In primary cell culture, TSHR activation substantially enhanced adipogenesis with 1.4 ± 0.07 (ORO), 8.6 ± 1.8 (foci), and 5.5 ± 1.6 (*LPL*) fold increases compared with controls. Surprisingly, TSHR activation in CM also significantly increased pre-BAT marker *PRDM16*; furthermore, TZD-ADM induced adipogenesis showed substantially increased BAT markers, *PGC-1α* and *UCP1*. Our study revealed that TSHR activation plays an important role in the adipogenesis process and BRITE/pre-BAT formation, which leads to WAT or BAT phenotype. It may contribute to weight loss as heat during hyperthyroidism and later transforms into WAT posttreatment of GD when patients gain excess weight.

## Introduction

Excess thyrotropin receptor (TSHR) activation occurs in two common conditions, Graves’ disease (GD) in which thyroid-stimulating antibodies (TSAB) mimic thyroid-stimulating hormone (TSH) causing hyperthyroidism and primary hypothyroidism when elevated circulating TSH compensates for low thyroid hormone (T4/T3) levels resulting from the failing gland (1). Both confer alterations in body composition, e.g., more than 90% of people with GD lose weight, mainly muscle mass and fat (2), while hypothyroidism increases fat and bone mineral density. The opposing differences of thyroid hormone levels have been traditionally suggested for these changes of body compositions, e.g., impact on white adipose tissue (WAT) or brown adipose tissue (BAT) metabolism (3, 4). BAT depots were thought to be absent from adult humans, but the availability of imaging using ^18^F-fluorodeoxyglucose positron emission tomography and computed tomography reveals their location in supraclavicular and neck regions (5–7).

Despite restoration of serum TSH concentrations to normal, many GD patients complain of substantial weight gain post treatment (8, 9) with potential negative impact on their future cardiovascular risk. Further studies in this area may therefore have considerable impact on determining the optimal treatment for patients with GD. There is still considerable controversy regarding the best treatment for Graves’ hyperthyroidism and radioiodine and/or thyroidectomy might be associated with more weight gain compared to those on antithyroid drugs and patients who undergo ablative therapy for thyroid cancer (10–12). This suggests that there are some factors associated with GD that influence post therapy weight gain. Furthermore, analysis has suggested that a diagnosis of GD (as opposed to other causes of thyroid over-activity) is an independent predictor of weight gain (10), raising the possibility that the persisting anti-TSH receptor antibodies in such patients might have long-term effects on peripheral adipose tissue composition (13). In humans, lipolysis was shown to be stimulated by TSH and TSAB, but this was confined to neonates suggesting an effect predominantly on BAT (14). Furthermore, the presence of functional extrathyroidal TSHR has been demonstrated in adipose tissue (15, 16) and bone (17), and fat-specific knockout of TSHR generated mice with larger adipocytes (18). More recent studies report a positive correlation between TSHR activation and obesity (19–21), and reports using animal models suggest a role for the TSHR in BAT and WAT function (22–24). For example, TSHR-deficient hyt/hyt mice became hypothermic in cold conditions, despite thyroxine administration, but transfection of TSHR into BAT of these mice improved core temperature (22). The above evidence led us to hypothesize that TSHR activation *per se* may contribute to changes in body composition separately from the effects of thyroid hormone levels, exerting a direct impact on adipose tissues metabolism (25).

Brown adipose tissue dissipates energy as heat (thermogenesis) in a process mediated by uncoupling protein 1 (UCP1), which uncouples oxidative phosphorylation from ATP production (26). WAT and BAT are derived from distinct lineages of mesenchymal stem cells (MSCs), Myf5+ for BAT (also muscle progenitors) but Myf5− for WAT (27). The two adipose types also differ morphologically with WAT having a single large fat vacuole and BAT having many smaller fat droplets and higher numbers of mitochondria (28). In addition to WAT and newly documented adult BAT (5), human beige (or BRITE for BRown in whITE) adipocytes have been recently identified, and like WAT are derived from Myf5− MSC (29). Although there are clearly defined BAT, BRITE, and WAT depots in mice, human fat depots tend to be heterogeneous with BRITE/pre-BAT adipocytes present in both WAT and inducible BAT depots with the potential to be transformed to either WAT or BAT (30, 31). Transcription factor *PRDM16* plays an essential role in the transformation of BRITE/pre-BAT to BAT (32). Adipocytes are generated by lineage-specific differentiation of MSC found in fat (33); the expression of TSHR is increased in human fat depots undergoing adipogenesis (34). We hypothesize that TSHR activation could thus modulate fat formation. Our aim was to investigate the effect of TSHR activation on human adipose tissue from the neck, which is recognized as a BAT inducible region (5), by phenotyping *ex vivo* samples using markers for WAT, BRITE, and BAT. Furthermore, we analyzed preadipocytes obtained from subcutaneous adipose tissue, to address the role of TSHR activation in adipogenesis and modulation of fat phenotype.

## Materials and Methods

All reagents were obtained from Sigma-Aldrich and tissue culture components from Cambrex unless otherwise stated.

### Adipose Tissue Collection

Subcutaneous adipose tissue (*n* = 16) was collected from patients undergoing elective open abdominal surgery for non-metabolic conditions. For *ex vivo* analysis, subcutaneous neck fat samples were obtained from GD (*n* = 11), toxic multinodular goiter (TMNG) (*n* = 6) and euthyroid control patients with benign thyroid nodules (*n* = 11) undergoing thyroid surgery. Five patients have undetectable TSH measurements (GD = 4 and TMNG = 1) with normal free T4 (one at upper limit normal level) or T3 levels. The suppressed TSH is expected in treated hyperthyroid patients as this will take months to recover despite being euthyroid. It should be stressed that all patients were clinically euthyroid during surgical procedure and patients’ information has been summarized in Table 1. All GD patients have positive TSHR antibodies measured by thyroid-binding inhibiting immunoglobulin assays and TSAB luciferase reporter assay (35).

**Table 1:** **Patients demographic**.

Patient ID	Sex	Age	Hist	FT3	FT4	TSH	TRAB	TPO	TSAB	EUT (months)
**GD**										
GD1	M	47	GD		13.0	2.06	32		2.6	8
GD2	F	71	GD	4.7	11.4	6.83	4.7	<2	2.9	7
GD3	F	23	GD		7.1	7.84	<1	1,059	3.0	7
GD4	M	48	GD	4.3	14.9	0.04	11.7	<2	2.4	4
GD5	F	63	GD		22.4	<0.02	6.7		2.5	3
GD6	F	39	GD	5.6	17.5	<0.02			3.1	6
GD7	F	52	GD	5.6	9.3	0.29	15.8	>1,000	3.0	21
GD8	F	38	GD		13.2	0.43			2.9	12
GD9	M	31	GD		12.3	2.34				3
GD10	F	57	TMNG	4.5	12.9	<0.02	19.3	648	2.7	10
GD11	F	27	TMNG	4.6	14.1	<0.02			3.3	6
**Toxic MNG**										
MNG1	F	43	TMNG	5.3	17.8	<0.02			1.6	2
MNG2	M	76	TMNG		13.0	0.1	<1		1.1	9
MNG3	F	61	TMNG		14.3	0.92		50	1.2	20
MNG4	F	70	TA		13.0	1.22	<1	12.5	1.2	16
MNG5	M	61	TMNG		13.5	0.21			1.2	21
MNG6	M	89	TMNG		14.1	0.25			1.1	9
**Control**										
CO1	F	21	CN		13.5	1.56			1.4	
CO2	F	78	HN		13.0	3.26			1.1	
CO3	F	46	BC		12.6	1.46			1.2	
CO4	F	71	EMNG		16.7	1.79			1.3	
CO5	M	50	EMNG		12.2	0.61			1.1	
CO6	F	27	EMNG		14.0	2.58		<2	1.2	
CO7	F	78	EMNG		13.7	0.11			1.8	
CO8	F	27	EMNG		14.6	1.09			1.2	
CO9	F	61	EMNG		14.0	0.57		<2	1.3	
CO10	M	45	EMNG		16.5	0.83		<2	1.4	
CO11	F	71	EMNG		13.0	0.65		300	1.2	

*F, female; M, male; Hist, histology; EUT, euthyroid duration; GD, Graves’ disease; TMNG, toxic multinodular goiter; EMNG, euthyroid multinodular goiter; TA, toxic adenoma; CN, colloid nodules; HN, hyperplastic nodules; BC, benign cyst*.

*Normal reference: FT3 (2.6–5.7 pmol/l), FT4 (9.2–22 pmol/l), thyroid-stimulating hormone (TSH) (0.30–4.40 mU/l), thyroid receptor antibodies (TRAB; <1 negative, 1–1.4, borderline >1.4 U/l positive), thyroid peroxidase antibodies (TPO) (<32 kU/l is negative), and thyroid-stimulating antibodies (TSAB; stimulation index 97.5th SD of normal <1.4 is negative)*.

### Generation of TSHR*-Expressing Cells

Preadipocyte/fibroblasts were obtained by collagenase digest, as previously described (36). Cells were used at low passage number (<5); hence, not all samples were analyzed in all experiments. Activating mutant TSHR (L629F) was introduced into the preadipocyte populations using retroviral vectors, previously produced in our laboratory (37). Geneticin selection resulted in mixed populations stably expressing the various TSHR, which exhibit increased basal levels of cAMP compared with the equivalent non-modified cell population, all as previously described (16).

### Preadipocyte/Fibroblast Culture *In Vitro* Adipogenesis

Preadipocytes were cultured in DMEM/F12 10% FCS (complete medium, CM). Adipogenesis was induced in confluent cells by replacing with differentiation medium [adipogenic medium (ADM)] containing 10% FCS, biotin (33μM), panthothenate (17μM), T3 (1nM), dexa-methasone (100nM), thiazolidinedione (TZD) (1μM), and insulin (500nM) for 22 days, adipogenesis was assessed by microscopic examination to detect the characteristic morphological changes (cell rounding, accumulation of lipid droplets), acquisition of lipid filled droplets [oil red O (ORO) staining], and transcript measurement of adipogenic markers (*PPAR*γ, *LPL*) by qPCR as described previously (16). In addition, foci of differentiation (groups of cells with lipid droplets) were counted in 10 different fields for each experimental condition (36).

### PCR Analysis of Markers for WAT, BRITE, or BAT

Transcript copy numbers for various genes, including markers for WAT [*LEPTIN* (38)], BRITE [*CITED1* (39)], pre-BAT (*PRDM16*), and BAT [*PGC-1α, UCP1*, and *ZIC1* (30, 31)] together with *TSHR* were measured.

Total RNA from cells or *ex vivo* fat tissues was extracted and reverse transcribed using standard protocols (16) for standard or qPCR analysis; primers (cross exon boundaries to avoid amplification of genomic DNA) were designed using primer 3 software (Table S1 in Supplementary Material). qPCR was conducted using SYBR Green incorporation measured on a Stratagene MX 3000. Comparison with plasmid standard curves for each gene permitted calculation of absolute values for each sample (transcripts per microgram input RNA). In addition, for qPCR, transcripts of a housekeeping gene, *APRT*, were measured so that values could be expressed relative to this (transcripts/1,000 *APRT*). *APRT* was also used in the comparative Ct method to assess transcript levels of *PRDM16*. It should be noted that none of the treatments used resulted in a variation in the *APRT* Ct value of more than one cycle. In qPCR experiments, all measurements were made in triplicate; the standard curve was also run in at least duplicate.

If multiple products (e.g., primer dimer) were detected by qPCR (dissociation curve), a classic PCR with densitometry technique was used. Standard PCR was performed to detect *CITED1* and *LEPTIN* using Phusion High-Fidelity PCR master mix (Thermo Scientific) as per the manufacturer’s instructions. The PCR products were resolved on 2% agarose gels for 35 min, and densitometry values were obtained and corrected to housekeeping gene (*GAPDH*).

### Statistical Analysis

Parametric data were analyzed using Student’s *t*-test and one-way ANOVA for multiple group comparisons where appropriate. Similarly, Mann–Whitney *U* test and Kruskal–Wallis *H* test was used for non-parametric data. All analysis was done using two-tailed tests. Parametric data were presented as mean ± SD and median ± interquartile range for non-parametric data. In all cases, *p* < 0.05 was considered significant.

## Results

### TSHR Activation Favor BRITE and WAT Formation in *Ex Vivo* Analysis

To examine the role of TSHR activation on adipose tissues, we analyzed markers of WAT, BRITE, and BAT using *ex vivo* samples of subcutaneous neck fat. Analyzed samples were obtained when patients were euthyroid, but the persisting TSAB result in GD fat samples experiencing ongoing TSHR activation while TMNG and control samples do not.

**Figure 1:**
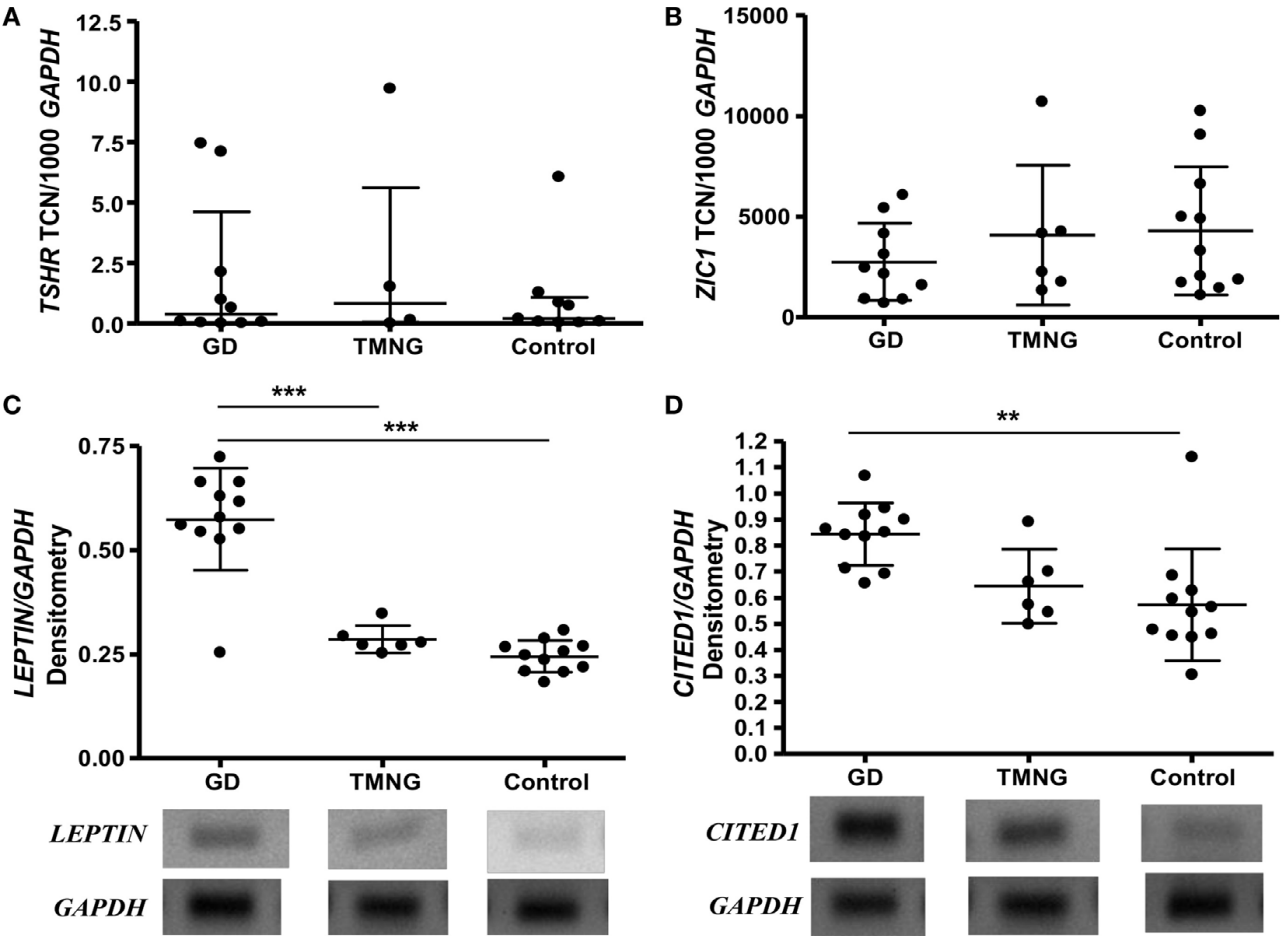
***Ex vivo* analysis of deep neck fat**. Samples were snap frozen, and total RNA was isolated. Gene transcripts were measured by qPCR, **(A)** thyrotropin receptor (*TSHR*); **(B)** *ZIC1* (brown adipose tissue marker), shown as transcript copy number (TCN) per 1,000 copies of housekeeper gene *APRT* (adenosine phosphoribosyl transferase); standard PCR was used to analyze **(C)** *LEPTIN* (white adipose tissue marker), **(D)** *CITED1* (BRITE marker), densitometry were measured and corrected to housekeeping gene (*GAPDH*). Representative photos were shown (gels with all samples had been included in Figure S1 in Supplementary Material). Post-ANOVA test for linear trend of *CITED1* was performed (*p* = 0.009). Results expressed as mean ± SD of all samples studied (each performed in duplicate) (**p ≤ 0.01; ***p < 0.001).

Expression levels of *TSHR* did not differ in control, TMNG, or GD groups (Figure 1A); we then analyzed the potential effect of TSAB/TSHR* on fat phenotype. Higher transcript levels of *LEPTIN* (WAT marker) were detected in GD samples compared to TMNG and control (*p* < 0.001) (Figure 1C), which indicates that adipogenesis in the WAT compartment is ongoing in GD. BAT markers *PGC-1α, UCP1*, and *ZIC1* were detected, even though there was no difference in expression levels between the three groups (Figure 1B). However, a well-defined BRITE marker, *CITED1*, showed highest transcript levels in GD samples, followed by TMNG and control (*p* value, test for trend = 0.0009) (Figure 1D).

These data suggest that TSHR activation is associated with WAT and BRITE fat generation; subsequently, we tried to understand the role of TSHR activation on adipogenesis using primary cell cultures.

### TSHR Activation Enhances Subcutaneous Adipogenesis Induced *In Vitro*

**Figure 2:**
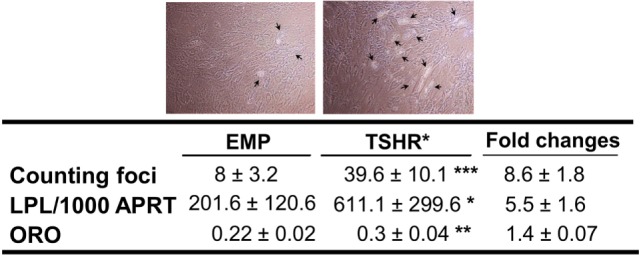
**Adipogenesis in subcutaneous preadipocytes was assessed by foci counting (*n* = 11, representative photos were shown with arrows indicating differentiating adipocytes), LPL transcripts measured by qPCR (*n* = 9) and oil red O (ORO, *n* = 4)**. Result presented as fold increase in TSHR* populations relative to empty vector controls. The table reports raw data for qPCR results expressed as transcript copy number per 1,000 copies of housekeeper gene *APRT* (adenosine phosphoribosyl transferase), together with foci numbers and ORO optical density values (mean ± SEM). Histograms = mean ± SEM of all samples studied (each performed in duplicate) (*p < 0.05; **p ≤ 0.01; ***p < 0.005).

We first investigated the effects of TSHR signaling on adipogenesis using subcutaneous preadipocytes stably expressing or not activating mutant TSHR (L629F, TSHR*). TSHR activation did not induce spontaneous adipogenesis in subcutaneous precursors even when the cells were examined for morphological signs up to 3 weeks after reaching confluence. In contrast, after 22 days incubation in an adipogenic cocktail, we observed substantial enhancement of this lineage-specific differentiation by TSHR activation, whether assessed morphologically, by semiquantitative ORO staining or qPCR measurement of transcripts for *LPL* (marker of terminal differentiation) as shown in Figure 2.

We concluded that TSHR activation enhances *in vitro*-induced adipogenesis. The adipogenic cocktail (ADM) used in this study contains PPARγ agonist TZD, which is known to stimulate BAT formation (40). Consequently, we conducted experiments to understand the impact of TSHR activation on BAT formation both in basal and induced adipogenesis conditions.

### TSHR Activation Enhanced BAT Formation of Subcutaneous Precursors

We selected several markers including pre-BAT *PRDM16*, BAT *PGC-1α* (transcriptional regulator of BAT formation), and *UCP1* (terminal BAT marker) (29).

These were measured in subcutaneous preadipocytes on day 0 in CM and following *in vitro*-induced adipogenesis in TZD-ADM for 22 days.

**Figure 3:**
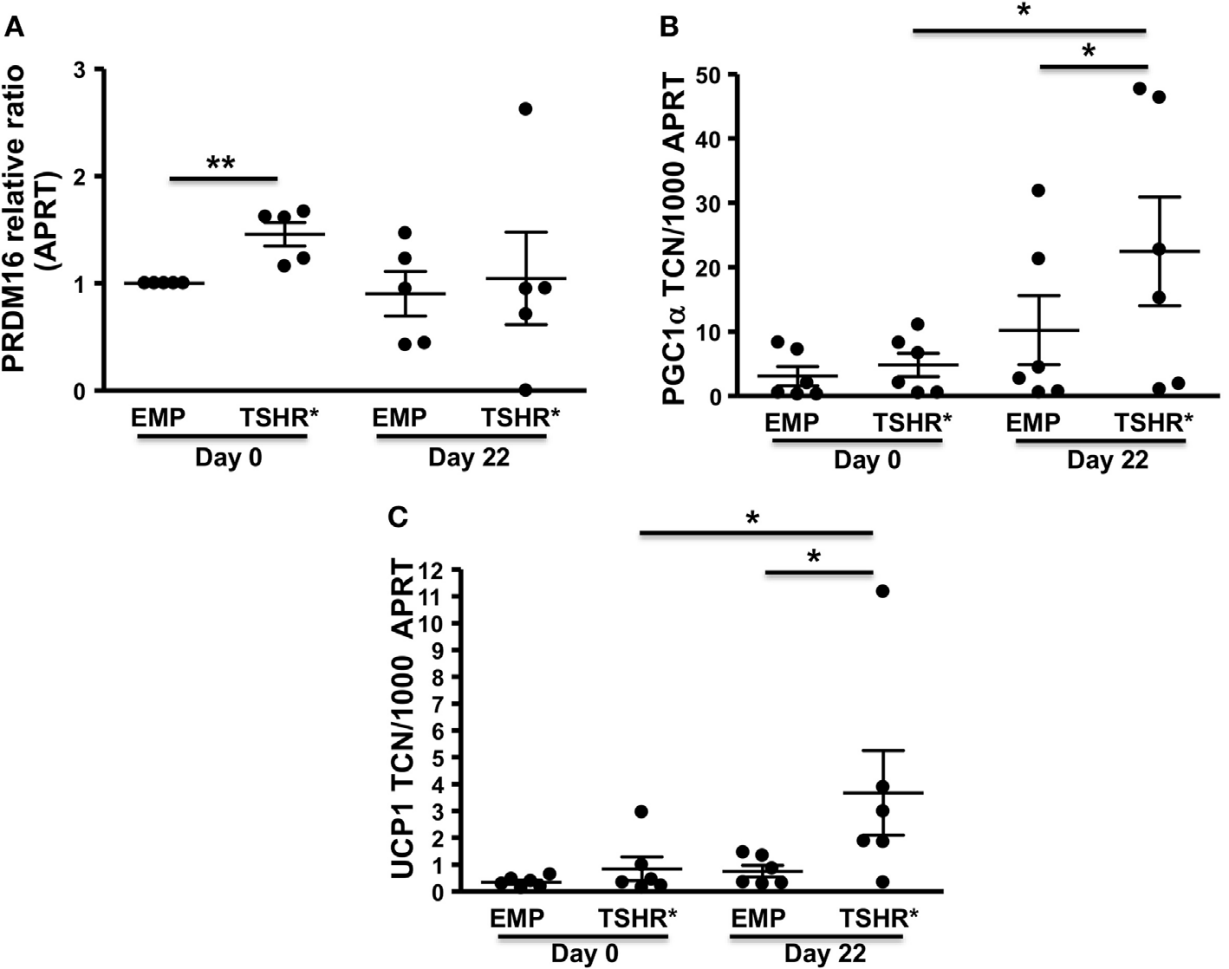
Preadipocytes/fibroblasts from subcutaneous fat (*n* = 6) expressing activating thyrotropin receptor (TSHR) mutant L629F (TSHR*) and equivalent empty vector controls were cultured until confluent (day 0) before changing to differentiation medium for 22 days. Total RNA was isolated before or after adipogenesis. PRDM16 (relative expression ratio) **(A)**, *PGC-1*α **(B)**, and uncoupling protein 1 (*UCP1*) **(C)** transcripts were measured by qPCR. Results are expressed as comparative qPCR (relative ratio to APRT) of PRDM16 or absolute qPCR (*PGC-1α* and *UCP1*) of transcript copy number (TCN) per 1,000 copies of housekeeper gene *APRT* (adenosine phosphoribosyl transferase). Histograms = mean ± SEM of all samples studied (each performed in duplicate). Two-tailed *t*-test or Mann–Whitney test used for statistic analysis (*p < 0.03; **p = 0.01).

On day 0 (basal condition), the cells experiencing TSHR activation displayed substantially higher transcript levels of the pre-BAT marker, *PRDM16*, when compared with the control population as shown in Figure 3A. However, TSHR activation had no significant effect on expression levels of *PGC-1α* and *UCP1* (Figures 3B,C).

By contrast, at day 22 following TZD-ADM induced adipogenesis, TSHR activation significantly increased transcript levels of *PGC-1α* and *UCP1* when compared with control cells in ADM conditions but lacking TSHR activation (Figures 3B,C).

## Discussion

Our study suggests that TSHR activation enhances adipogenesis and could contribute to the modulation of fat phenotype.

The *ex vivo* data demonstrate that in samples of fat from TSAB positive GD patients, transcript levels of markers for WAT (*LEPTIN*) and BRITE (*CITED1*) were significantly higher than in corresponding samples from people with TMNG or controls (all TSAB negative). Since all patients were euthyroid at the time of surgery, it is reasonable to conclude that the TSAB have a role in the observed effect and the presence of TSHR transcripts in all samples confirms that this would be plausible.

Having access to samples of neck adipose tissue is fortuitous since Cypess and colleagues reported a gradient from the surface to the midline of WAT *via* BRITE to BAT in this region using biomarkers, e.g., *LEPTIN* (WAT), gradient *UCP1* expression (BRITE to BAT) or *ZIC* (41, 42). Studies in mice suggest a bidirectional interconversion of BRITE and white adipocytes (43), with BRITE adipocytes being induced by cold temperature into brown fat and conversely returning into white fat at higher temperatures. Similarly, Lee et al. found that BRITE fat can be transformed into brown or white by adrenergic stimulation and high-calorie diet, respectively (44). High thyroid hormone levels are known to induce brown fat activity in BAT and BRITE fat (3, 4, 45). Of note, the samples from the GD and TMNG patients would have previously encountered a period of thyroid hormone excess, during the hyperthyroid phase of their condition but only in GD would there have been simultaneous TSHR activation.

We hypothesize that in GD, TSHR activation increases adipogenesis and, combined with excess thyroid hormone, favors formation of BAT. This could also explain the heat intolerance of GD patients, which is usually attributed to excess thyroid hormone increasing metabolic rate. The concept is further supported by studies in the hyt/hyt mouse, which lacks a functional TSHR and deals poorly with low temperature, a characteristic which can be overcome by transfecting WT TSHR into the animals (22). Proof could be provided by comparing expression of WAT, BRITE, and BAT markers in adipose tissue from TSAB-positive GD patients when hyperthyroid and then when euthyroidism is restored; however, patient safety during surgery precludes this.

Our *in vitro* studies confirmed that TSHR activation increases adipogenesis in subcutaneous fat, in contrast to orbital fat, in which we have previously reported that TSHR activation inhibits induced adipogenesis (16). These effects are the opposite of the situation in hyperthyroid GD in which the majority of fat stores are depleted, with the exception of orbital fat which expands in some patients. Our studies have demonstrated that orbital adipogenesis is under differing regulatory mechanisms compared with non-orbital fat precursors (46, 47).

We also investigated whether TSHR activation had any effect on fat phenotype *in vitro*, we recognize that there may be some overlap in markers for WAT, BAT, pre-BAT, and BRITE but have selected the best characterized for each (48). We found that subcutaneous precursors, experiencing TSHR activation, had significantly higher transcript levels of the pre-BAT marker, *PRDM16* in basal conditions. There were no other indicators of spontaneous adipogenesis in these cells. In ADM, TSHR activation significantly increased differentiation and also significantly increased expression levels of BAT markers *PGC*-1 and *UCP1*. Our findings are similar to those of Cypess et al. who induced adipogenesis in the presence of Db-cAMP and obtained significant enhancement of *PGC-1α* and *UCP1* compared with control cells in ADM alone (41). In our *in vitro* model, cells experiencing TSHR activation (mutant TSHR) display a twofold to fourfold increase in basal cAMP when compared to empty vector control cells (16).

The *in vitro* findings support our hypothesis that TSHR activation enhances adipogenesis and favors BAT formation in the hyperthyroid state. Our *ex vivo* findings imply that enhanced adipogenesis persists but the fat phenotype is WAT with some features of BRITE rather than BAT. Could this contribute to the weight gain experienced in GD following treatment? If increased adipogenesis produces BAT, energy can be dissipated as heat (as in GD) and weight will be shed. Once the BAT phenotype is lost (posttreatment of GD), then the increased adipogenesis would lead to WAT accumulation and weight will be gained.

Current therapy is based on inhibiting thyroid hormone production medically, surgically, or using radioiodine ablation. Future strategies aimed at neutralizing TSAB, for example, using TSHR antagonist could be more effective in solving weight problem.

## Ethics Statement

The South East Wales Research Ethics committee approved this study; all fat samples were collected with informed consent, and written consent was obtained.

## Author Contributions

LZ and MD performed majority of the experiments; LZ, ML, and MD wrote the manuscript. DR and CD obtained ethical approval and reviewed the manuscript. MS, DS-C, and MD obtained fat samples from patients. LZ and ML coordinated the project, data analysis, and writing of the manuscript.

## Conflict of Interest Statement

The authors declare that the research was conducted in the absence of any commercial or financial relationships that could be construed as a potential conflict of interest.

## Abbreviations

TSHR, thyrotropin receptor; MSC, mesenchymal stem cell; TSAB, thyroid-stimulating antibodies; CM, complete medium; ADM, adipogenic medium; WAT, white adipose tissue; BAT, brown adipose tissue.
