# Supplementary material for: The Role of Thyrotropin Receptor Activation in Adipogenesis and Modulation of Fat Phenotype
Source: Front Endocrinol (Lausanne). 2017 Apr 19;8:83. doi: 10.3389/fendo.2017.00083 (PMC5395630; doi:10.3389/fendo.2017.00083)
Supplement: Supplementary file 1 [file image_1.pdf]

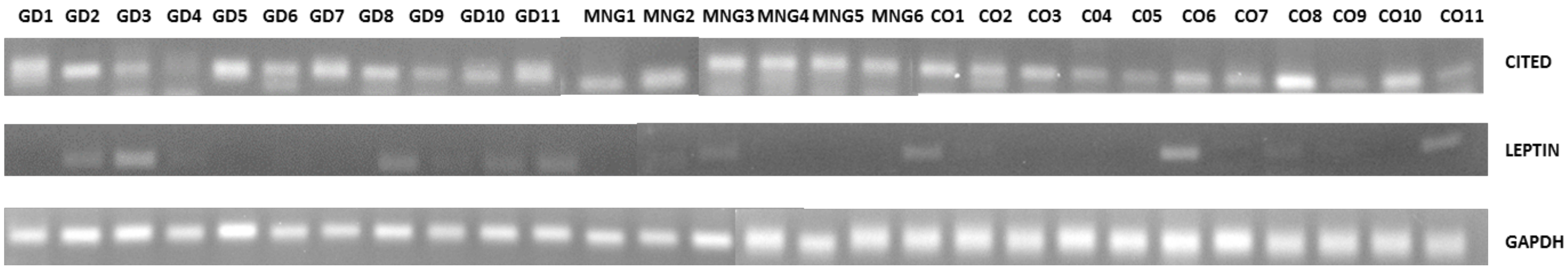

Supplementary figure 1: Agarose gel (2%) in 1xTAE confirming the size of the *CITED* PCR product of 117bp, *LEPTIN* PCR product of 158 bp and *GAPDH* PCR product 156 bp. Samples tested: GD# represents Graves' disease patient, MNG# represents multinodular goitre and CO# represents control ("#" number of the sample).
