# Supplementary material for: The Role of Thyrotropin Receptor Activation in Adipogenesis and Modulation of Fat Phenotype
Source: Front Endocrinol (Lausanne). 2017 Apr 19;8:83. doi: 10.3389/fendo.2017.00083 (PMC5395630; doi:10.3389/fendo.2017.00083)
Supplement: Supplementary file 2 [file table_1.pdf]

***Supplemental Table 1. QPCR primers used in this study indicating exon location.***

| Gene | Forward Primer | Reverse Primer |
|---|---|---|
| h*APRT* | GCTGCGTGCTCATCCGAAAG (Exon 3) | CCTTAAGCGAGGTCAGCTCC (Exon 5) |
| h*LPL* | GAGATTTCTCTGTATGGACC (Exon 7) | CTGCAAATGAGACACTTTCTC (Exon 9) |
| h*ZIC1* | CCCTTCAAGTGCGAGTTTGA (Exon 2) | TGGACCTTCATGTGTTTGCG (Exon 3) |
| h*PRDM16* | AGAGATTCCGCGAGCCGACACCAT (Exon 1) | CTCCCCGGCTTCCATCTTCCTCTT (Exon 2) |
| h*PGC-1α* | GAAGAGCGCCGTGTGATTTA (Exon 10-11) | CGCTGTCCCATGAGGTATTC (Exon 13) |
| h*UCP-1* | GGGGCTTCAGCGGCAAATCAG (Exon 2) | TATAAGTCCCCGTGTAGCGAGGTT (Exon 3) |
| h*CITED1* | CTCACCTGCGAAGGAGGATG (Exon 2) | CCATTTGAGGCTACCCCAGG (Exon 3) |
| h*Leptin* | AATGCATTGGGGAACCCTGT (Exon 2) | AGGAGACTGACTGCGTGTGT (Exon 3) |
| h*GAPDH* | ATGCCATCACTGCCACCCAGA (Exon 5) | TGCCAGTGAGCTTCCCGTTCA (Exon 6) |
